# ZIFA: Dimensionality reduction for zero-inflated single-cell gene expression analysis

**DOI:** 10.1186/s13059-015-0805-z

**Published:** 2015-11-02

**Authors:** Emma Pierson, Christopher Yau

**Affiliations:** Department of Statistics, University of Oxford, 1 South Parks Road, OX1 3TG, Oxford, UK; Wellcome Trust Centre for Human Genetics, University of Oxford, Roosevelt Drive, OX3 7BN, Oxford, UK

## Abstract

Single-cell RNA-seq data allows insight into normal cellular function and various disease states through molecular characterization of gene expression on the single cell level. Dimensionality reduction of such high-dimensional data sets is essential for visualization and analysis, but single-cell RNA-seq data are challenging for classical dimensionality-reduction methods because of the prevalence of dropout events, which lead to zero-inflated data. Here, we develop a dimensionality-reduction method, (Z)ero (I)nflated (F)actor (A)nalysis (ZIFA), which explicitly models the dropout characteristics, and show that it improves modeling accuracy on simulated and biological data sets.

**Electronic supplementary material:**

The online version of this article (doi:10.1186/s13059-015-0805-z) contains supplementary material, which is available to authorized users.

## Introduction

Single-cell RNA expression analysis (scRNA-seq) is revolutionizing whole-organism science [1, 2] allowing the unbiased identification of previously uncharacterized molecular heterogeneity at the cellular level. Statistical analysis of single-cell gene expression profiles can highlight putative cellular subtypes, delineating subgroups of T cells [3], lung cells [4] and myoblasts [5]. These subgroups can be clinically relevant: for example, individual brain tumors contain cells from multiple types of brain cancers, and greater tumor heterogeneity is associated with worse prognosis [6].

**Fig. 1:**
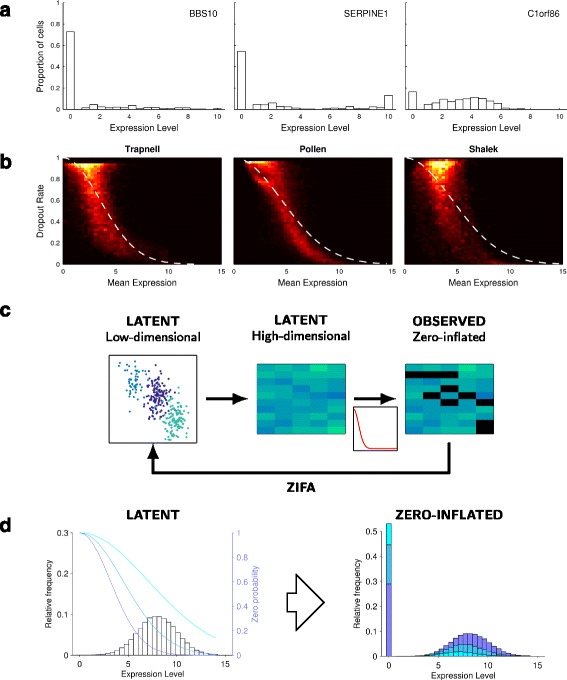
Zero-inflation in single-cell expression data. **a** Illustrative distribution of expression levels for three randomly chosen genes showing an abundance of single cells exhibiting null expression [15]. **b** Heat maps showing the relationship between dropout rate and mean non-zero expression level for three published single-cell data sets [3, 5, 14] including an approximate double exponential model fit. **c** Flow diagram illustrating the data generative process used by ZIFA. **d** Illustrative plot showing how different values of *λ* in the dropout-mean expression relationship (*blue lines*) can modulate the latent gene expression distribution to give a range of observed zero-inflated data

Despite the success of early single-cell studies, the statistical tools that have been applied to date are largely generic, rarely taking into account the particular structural features of single-cell expression data. In particular, single-cell gene expression data contain an abundance of dropout events that lead to zero expression measurements. These dropout events may be the result of technical sampling effects (due to low transcript numbers) or real biology arising from stochastic transcriptional activity (Fig. 1a). Previous work has been undertaken to account for dropouts in univariate analysis, such as differential expression analysis, using mixture modeling [7, 8]. However, approaches for multivariate problems, including dimensionality reduction, have not yet been considered. As a consequence, it has not been possible to ascertain fully the ramifications of applying dimensionality-reduction techniques, such as principal components analysis (PCA), to zero-inflated data.

Dimensionality reduction is a universal data-processing step in high-dimensional gene expression analysis. It involves projecting data points from the very high-dimensional gene expression measurement space to a low-dimensional *latent* space reducing the analytical problem from a simultaneous examination of tens of thousands of individual genes to a much smaller number of (weighted) collections that exploit gene co-expression patterns. In the low-dimensional latent space, it is hoped that patterns or connections between data points that are hard or impossible to identify in the high-dimensional space will be easy to visualize.

The most frequently used technique is PCA, which identifies the directions of largest variance (principal components) and uses a linear transformation of the data into a latent space spanned by these principal components. The transformation is linear as the coordinates of the data points in the low-dimensional latent space are a weighted sum of the coordinates in the original high-dimensional space and no non-linear transformations are used. Other linear techniques include factor analysis (FA), which is similar to PCA but focuses on modeling correlations rather than covariances. Many non-linear dimensionality techniques are also available but linear methods are often used in an initial step in any dimensionality-reduction processing since non-linear techniques are typically more computationally complex and do not scale well to simultaneously handling many thousands of genes and samples.

In this article, we focus on the impact of dropout events on the output of dimensionality-reduction algorithms (principally linear approaches) and propose a novel extension of the framework of probabilistic principal components analysis (PPCA) [9] or FA to account for these events. We show that the performance of standard dimensionality-reduction algorithms on high-dimensional single-cell expression data can be perturbed by the presence of zero-inflation making them suboptimal. We present a new dimensionality-reduction model, zero-inflated factor analysis (ZIFA), to account explicitly for the presence of dropouts. We demonstrate that ZIFA outperforms other methods on simulated data and single-cell data from recent scRNA-seq studies.

The fundamental empirical observation that underlies the zero-inflation model in ZIFA is that the dropout rate for a gene depends on the expected expression level of that gene in the population. Genes with lower expression magnitude are more likely to be affected by dropout than genes that are expressed with greater magnitude. In particular, if the mean level of non-zero expression (log read count) is given by *μ* and the dropout rate for that gene by *p*_0_, we have found that this dropout relationship can be approximately modeled with a parametric form *p*_0_= exp(−*λμ*^2^), where *λ* is a fitted parameter, based on a double exponential function. This relationship is consistent with previous investigations [7] and holds in many existing single-cell data sets (Fig. 1b), including a data set with unique molecular identifiers [10] (Additional file 1: Figure S1). The use of this parametric form permits fast, tractable linear algebra computations in ZIFA enabling its use on realistically sized data sets in a multivariate setting.

## Method

### Overview

ZIFA adopts a latent variable model based on the FA framework and augments it with an additional zero-inflation modulation layer. Like FA, the data generation process assumes that the separable cell states or subtypes initially exist as points in a latent (unobserved) low-dimensional space. These are then projected onto points in a latent high-dimensional gene expression space via a linear transformation and the addition of Gaussian-distributed measurement noise. Each measurement then has some probability of being set to zero via the dropout model that modulates the latent distribution of expression values. This allows us to account for observed zero-inflated single-cell gene expression data (Fig. 1c). The scaling parameter in the dropout model can allow for a large range of dropout-expression profiles (Fig. 1d).

In the following, we provide a more detailed mathematical treatment of the proposed zero-inflated factor analysis model, although we leave a complete exposition for Additional file 1. A Python-based software implementation and source code are made freely available online via an MIT License: https://github.com/epierson9/ZIFA.

### Statistical model

Let *N* be the number of samples, *D* be the number of genes and *K* be the desired number of latent dimensions. The data are given by a high-dimensional *N*×*D* data matrix **Y**= [**y**_1_,…,**y**_*N*_], where *y*_*ij*_ is the level of expression (log read count) of the *j*th gene in the *i*th sample. The data are assumed to be generated from a projection of a latent low-dimensional *N*×*K* matrix **Z**= [**z**_1_,…,**z**_*N*_] (*K*≪*D*). In all derivations below, we use use *i*=1,…,*N* to index over samples (cells), *j*=1,…,*D* to index over genes and *k*=1,…,*K* to index over latent dimensions. Each sample **y**_*i*_ is drawn independently:

(1) $$\begin{array}{@{}rcl@{}} \textbf{z}_{i} & \sim &\text{Normal}(0, \textbf{I}), \end{array} $$

(2) $$\begin{array}{@{}rcl@{}} \textbf{x}_{i} | \textbf{z}_{i} & \sim& \text{Normal}(\textbf{A} \textbf{z}_{i} + \boldsymbol{\mu}, \textbf{W}), \end{array} $$

(3) $$\begin{array}{@{}rcl@{}} h_{ij} | x_{ij} & \sim &\text{Bernoulli}(p_{0}), \end{array} $$

(4) $$\begin{array}{@{}rcl@{}} y_{ij} & =& \left\{ \begin{array}{ll} x_{ij}, & \text{if}\ h_{ij} = 0, \\ 0, & \text{if}\ h_{ij} = 1, \end{array} \right. \end{array} $$

where **I** denotes the *K*×*K* identity matrix, **A** denotes a *D*×*K* factor loadings matrix, **H** is a *D*×*N* masking matrix, $\textbf {W} = \text {diag}\left ({\sigma _{1}^{2}}, \dots, {\sigma _{D}^{2}}\right)$ is a *D*×*D* diagonal matrix and ***μ*** is a *D*×1 mean vector. We choose the dropout probability to be a function of the latent expression level, $p_{0} = \text {exp}\left (-\lambda x_{\textit {ij}}^{2}\right)$, where *λ* is the exponential decay parameter in the zero-inflation model. Note that *λ* is shared across genes, which reduces the number of parameters to be estimated and captures that technical noise should have similar effects across genes.

### Statistical inference

Given an observed single-cell gene expression matrix **Y**, we wish to identify model parameters *Θ*=(*A*,*σ*^2^,*μ*,*λ*) that maximize the likelihood *p*(**Y**|*θ*). We do this using the expectation-maximization (EM) algorithm. We summarize the algorithm in the box below and then describe the algebraic details:



We denote the value of the parameters at the *n*th iteration, *Θ*_*n*_, as the value that maximizes the expected value of the complete log likelihood *p*(**Z**,**X**,**H**,**Y**) under the conditional distribution over the latent variables given the observed data and the parameters at the last iteration. Computing the value of the parameters at each iteration requires two steps: the expectation step (E-step) and the maximization step (M-step). In the E-step, we derive an expression for the complete log likelihood *p*(**Z**,**X**,**H**,**Y**|*Θ*_*n*_) and compute all necessary expectations under the distribution *p*(**Z**,**X**,**H**|**Y**,*Θ*_*n*−1_). The approximate zero-inflation model that we adopt admits closed form expressions for the expectations allowing the algorithm to be applied to realistically sized data sets. In the M-step, we maximize the expected value of the complete log likelihood with respect to *Θ*_*n*_.

The EM algorithm structurally resembles the equivalent algorithm for FA that iterates between imputing the coordinates of the observed data points in the low-dimensional latent space (E-step) and optimizing model parameters (M-step). In ZIFA, the expectation step incorporates a data imputation stage to compute the expected gene expression levels for genes/cells with observed null values. Note that if the noise measurement variances attributed to each gene are identical, we obtain a zero-inflated version of the probabilistic PCA algorithm [9] (ZI-PPCA).

### Fast approximation for whole transcriptome analysis

**Fig. 2:**
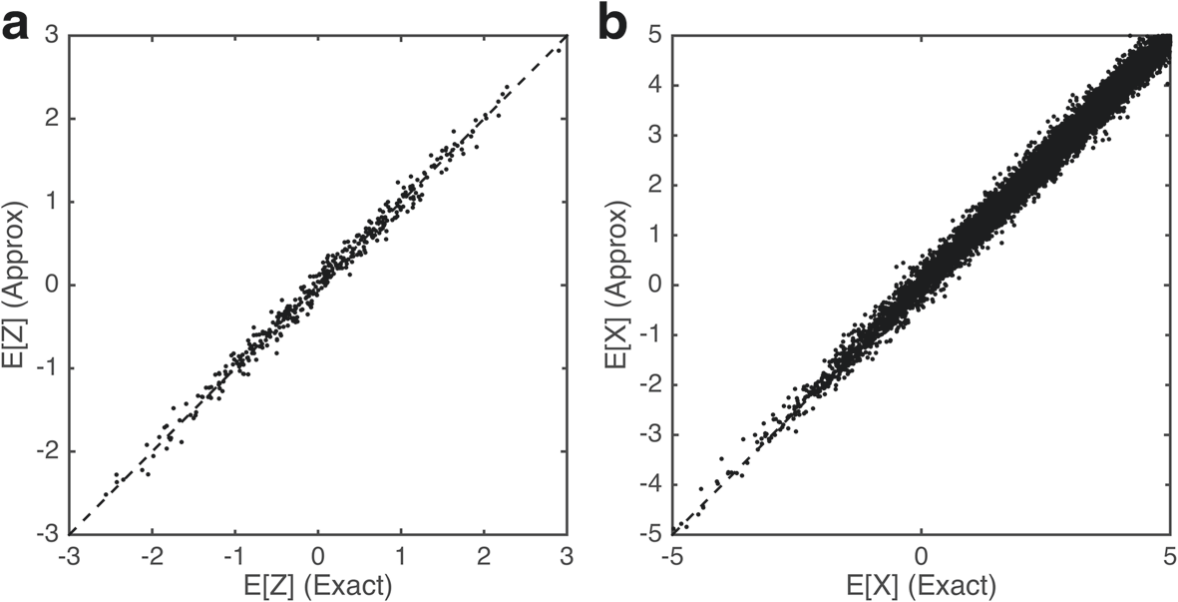
Comparison of exact and block-based EM algorithms. Plots show the correlation between expectations computed using the exact and block-based EM algorithms for latent low-dimensional positions (**Z**) (**a**) and latent observations **X** (**b**). Simulations were performed on a simulated data set with 500 genes and 200 cells. A block size of 50 was chosen for the approximate approach

The EM algorithm requires computations involving conditional expectations of multivariate Gaussian distributions. For each cell, information from non-zero measurements is used to impute the expected expression levels for genes with zero measured values *jointly*. If all available expressed genes are used for this imputation process, the *exact* computations would necessitate large computationally intensive matrix multiplications. In practice, we have discovered that it is not necessary to compute the expectations using all available genes at once. Substantial computational savings can be achieved by partitioning the genes into non-overlapping disjoint sets, and then performing exact computations within each block of genes. This decreases the run time of our algorithm from quadratic to linear in the number of genes, allowing it to run on data sets with hundreds of samples and tens of thousands of genes on a standard computer. Figure 2 shows that expectations obtained via this approximate strategy closely follow those from exact calculations but can be achieved with a substantial computational speed-up. Parameter estimates based on these approximate expectations are also robust (Additional file 1: Figure S2).

Table 1 details running times using our serial Python implementation for four data sets. For all data sets, we filtered out genes that were zero more than 95 % of the time except for the 11 cell populations data set, for which the algorithm did not converge unless we filtered out genes that had zeros across more than 80 % of samples. Tests were run on a standard quad-core Apple MacBook Pro laptop computer. We do not report timings for the exact version of our algorithm as these require many orders of magnitude more compute time. The computational times are not of the order of seconds, like PCA or FA, as a price must be paid for the increased expressive power of ZIFA. However, the availability of exact and approximate versions of ZIFA does allow the application of the method for data of a variety of sizes. The computational implementation of our approximate inference method can also be parallelized since the expectation calculations are independent across each cell and gene subset. We seek to implement this strategy in future versions of the software.

**Table 1:** Computational times for single-cell data sets of various sample and gene set sizes using the approximate version of our method

Data set	# Samples	# Genes	Run time (mins)
Differentiating T cells [3]	182	8.968	4.5
Myoblasts [5]	372	15,529	26.7
Bone marrow [14]	1861	11,115	61.0
11 Populations [15]	249	12,336	9.9

## Results

### Simulation study

We tested the relative performance of ZIFA against PCA, PPCA [9], FA and, for reference, non-linear techniques including stochastic neighbor embedding (t-SNE) [11], Isomap [12] and multidimensional scaling [13]. First, we generated simulated data sets according to the PPCA/FA data generative model with the addition of one of three dropout models: (i) a double exponential model (as assumed by ZIFA), (ii) a linear decay model and (iii) a missing-at-random uniform model. The latter two models were designed to test the robustness of ZIFA to extreme misspecification of the dropout model. Data were simulated under a range of different conditions by varying noise levels, dropout rates, number of latent dimensions and number of genes. The simulation experiment was not intended to truly reflect actual real world data characteristics but to establish, when all other modeling assumptions are true, the impact of dropout events on the outcomes of (P)PCA and FA.

#### Setup

We used the assumed generative model to produce simulated data. For the simulations, the values *a*_*jk*_ were drawn from a uniform distribution *U*(−0.5,0.5), the diagonal elements of the covariance matrix were drawn from a uniform distribution *U*(0.9,1.1)*σ*^2^, where *σ*^2^ is a simulation parameter, and *μ*_*j*_ were drawn from *U*(2.7,3.3). We experimented with three choices of *f*(·): a decaying squared exponential, $f(X_{\textit {ij}}) = \exp \left (-\lambda X_{\textit {ij}}^{2}\right)$ (used in ZIFA); a linear decay function, *f*(*X*_*ij*_)=1−*λX*_*ij*_; and a uniform (missing at random) function for each gene *j*, *f*(*X*_*ij*_)=1−*λ*_*j*_.

We used a base setting of *N*=150, *K*=10, *D*=50, *σ*^2^=0.3 and *λ*=0.1, and we explored the effects of altering the decay parameter *λ*, the number of latent dimensions *K*, the cluster spread *σ*^2^, the number of observed dimensions *D* and the number of samples *N*.

#### Performance metrics

As a measure of algorithm performance, we compared the true **z**_*i*_ to the $\hat {\mathbf {z}}_{i}$ for each sample estimated by the algorithms as follows. We computed the true distance between each pair of points *j,k* and defined a pairwise distance matrix *F* such that *F*_*jk*_=||**z**_*j*_−**z**_*k*_||_2_. We compared this to the estimated distance matrix $\hat F_{\textit {jk}} = ||\hat {\textbf {z}}_{j} - \hat {\textbf {z}}_{k}||_{2}$. We scored the correspondence between the two distance matrices using the Spearman correlation. By comparing *F* and $\hat F$ rather than **z**_*i*_ and $\hat {\textbf {z}}_{i}$, we account for the fact that dimensionality-reduction algorithms may rotate the points but ought to preserve the relative distances between them.

#### Outcomes

**Fig. 3:**
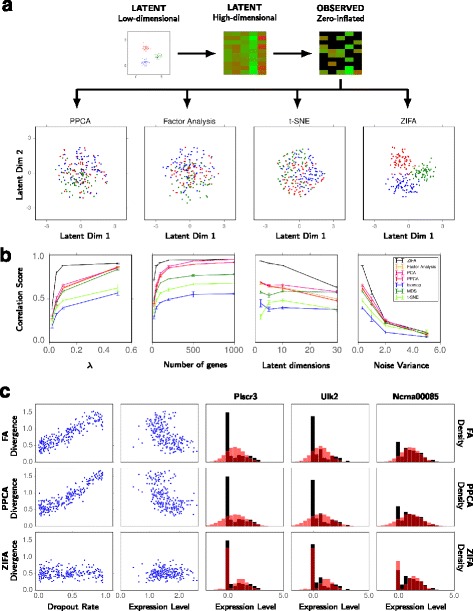
Performance comparison of dimensionality-reduction techniques. **a** Toy simulated data example illustrating the performance of ZIFA compared to standard dimensionality-reduction algorithms. **b** Performance on simulated data sets based on correlation score between the estimated and true latent distances as a function of *λ* (larger *λ*, lower dropout rate), number of genes and latent dimensions, and noise level used in the simulations. **c** Plots showing the divergence between the predictive and empirical data distributions as a function of dropout rate and mean expression level for FA, PPCA and ZIFA. Illustrative predictive performance and model fits (*red*, color online) on the T-cell single-cell data set (*black*) [3]

Although the data sets were generated according to a PPCA/FA model (up to the dropout stage), in the presence of cells with genes possessing zero expression, the performance of all standard dimensionality-reduction methods (even PPCA/FA) deteriorated relative to ZIFA. An example is illustrated in Fig. 3a. Our simulation results (Fig. 3b) indicate that standard approaches may be safely used in certain regimes but should be avoided in others. In particular, gene sets with a high degree of zero-inflation will be problematic (small *λ*), as the relative distances between data points in the gene expression measurement space will be distorted by the presence of zeros and hence there will be an error when projecting back into the latent space. Performance also falls if the gene set is small since there is less scope to exploit strong co-expression signatures across genes to mitigate for the presence of zeros. These regimes are important to consider in the context of linear transformation techniques (PCA, PPCA and FA) that are often applied only to curated gene sets where the linearity constraints may be approximately applicable. The application of non-linear techniques did not cure the problems induced by dropouts.

Overall, ZIFA outperformed the standard dimensionality-reduction algorithms. This would be expected for those simulations adopting the same generative model assumed by ZIFA (Fig. 3b) but performance was also replicated regardless of whether dropouts were added following a linear model (Additional file 1: Figure S3A), or a missing-at-random model (Additional file 1: Figure S3B). This suggests that it is better to account for dropouts somehow even if the dropout characteristics are not realistic. Interestingly, this may suggest that ZIFA could be applicable for other zero-inflated multivariate data sets. Additional file 1: Figure S4 also shows performance measured in terms of sum-of-squared error rather than Spearman correlation.

ZIFA should, therefore, be considered a safe alternative in that it converges in performance to PPCA/FA in the large-data low-noise limit but is robust to dropout events that might distort the outcomes of these methods in non-ideal situations.

### Single-cell data modeling

We next sought to test these methods in an experiment based on real single-cell expression data sets [3, 5, 6, 14]. In this case, the true latent space is unknown and we are unable to measure performance as with the previous simulated data experiment. Instead, for each of the data sets, we took random subsets of 25, 100, 250 and 1000 genes and applied ZIFA, PPCA and FA to each subset assuming five latent dimensions.

For each gene *j*, we compared the posterior predictive distribution $\hat Y_{j}$ of the distribution of read counts from each method to the observed distribution *Y*_*j*_ as follows: (1) we computed the proportion of values in *Y*_*j*_ and $\hat Y_{j}$ that fell into 30 discrete intervals, (2) we then computed the difference between the histograms *Δ*_*j*_. If *h*_*n*_ is the proportion of values in bin *n* for the true distribution, and $\hat h_{n}$ for the predicted distribution, then the histogram divergence is given by

(5) $$ \Delta_{j} = \sum_{n = 1}^{30} |h_{n} - \hat h_{n}|.  $$

We computed the fraction of genes for which the *Δ*_*j*_ from ZIFA was less than *Δ*_*j*_ from PPCA and FA. To prevent overfitting, we assessed the fit on a test set: we fitted the model for each data set on a training set containing 70 % of the data points, and computed the difference between the histograms on the remaining 30 % of data points.

Note that it is not possible to do this comparison with standard PCA or other dimensionality methods, such as t-SNE, since these are not based on a probabilistic generative model framework and therefore, it is not possible to derive the posterior predictive distributions that we use for performance comparisons.

Using this criterion, we found that predictive distributions from PPCA and FA showed high divergence for genes that exhibited a high dropout rate or possessed a low non-zero expression level. This meant that the predictive data distributions were a poor fit for the empirical data. The performance of ZIFA was largely unaffected in contrast (Fig. 3c). Example predictive model fits are shown for the T-cell data set [3] for three genes: Plscr3, Ulk2 and Ncrna00085 (Fig. 3c).

The statistical frameworks underlying PPCA and FA employ Gaussianity assumptions that are unable to account explicitly for zero-inflation in single-cell expression data. The dropout model used by ZIFA modulates this Gaussianity assumption allowing for zero-inflation leading to drastically improved modeling accuracy. Across the four data sets, we found that the predictive distribution derived by ZIFA was superior to those of PPCA and FA on at least 80 % of the genes examined and often over 95 % (Table 2).

**Table 2:** Comparison of ZIFA to PPCA and FA on four biological data sets

Data set	Method	Subset size			
		25	100	250	1000
Differentiating T cells [3]	FA	86±6.6 %	84±4.6 %	82±4.9 %	84±8.8 %
	PPCA	88±6.3 %	87±4.1 %	89±4.5 %	100±0.3 %
11 populations [15]	FA	97±3.7 %	96±2.5 %	96±2.1 %	95±2.7 %
	PPCA	98±3.2 %	97±2.0 %	97±1.5 %	99±0.6 %
Myoblasts [5]	FA	97±3.3 %	97±2.4 %	96±2.7 %	95±2.7 %
	PPCA	97±3.2 %	96±2.3 %	96±2.1 %	99±1.7 %
Bone marrow [14]	FA	98±3.0 %	97±2.0 %	97±1.7 %	97±1.7 %
	PPCA	98±3.1 %	97±1.8 %	97±1.4 %	97±1.3 %

The column headings are the number of genes in the data set (selected at random). Percentages denote the proportion of genes for which ZIFA provided a better fit than FA/PPCA, averaged across 100 replicates

**Fig. 4:**
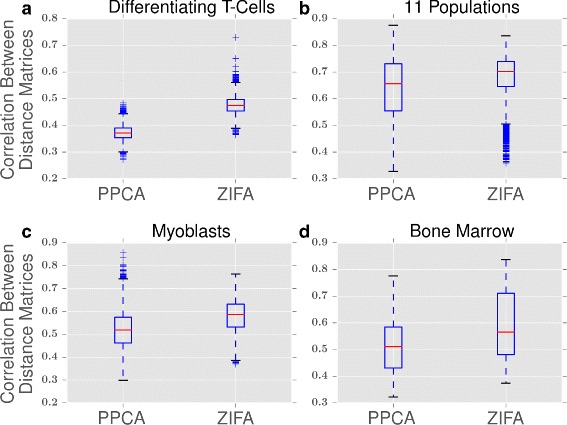
Consistency of cell-to-cell distances. Box plots showing the correlation between distance matrices for PPCA and ZIFA from 100 gene sets selected at random from (**a**) differentiating T cells [3], (**b**) 11 populations [15], (**c**) myoblasts [5] and (**d**) bone marrow [14]. The distance matrices produced by ZIFA are more correlated with each other than are the distance matrices produced by PPCA

We further assessed whether the low-dimensional projections by ZIFA were more consistent than those of PPCA. For the four data sets, we repeated the following procedure 100 times: we sampled 100 genes at random, ran ZIFA or PPCA, and computed the pairwise distances between points in the low-dimensional space. This yielded 100 distance matrices, one for each iterate. We computed the Spearman correlation between each pair of distance matrices (for a total of 100×99/2 correlations) and recorded the average Spearman correlation for both ZIFA and PPCA. Figure 4 shows the distribution of the Spearman correlations for ZIFA and PPCA on the four data sets. Overall, the distance matrices produced by ZIFA were more correlated with each other than those produced by PPCA, indicating that the ZIFA distance matrices are more consistent across random iterates as ZIFA’s performance is less dependent on the number of dropout events present in the data.

### Cell type separability

We now address the utility of ZIFA for a common analytical problem in single-cell expression analysis: the identification of distinct cellular subtypes or states. Typically, this occurs by reducing the high-dimensional gene expression measurements to a low-dimensional representation (often with PCA). The data are then clustered in this low-dimensional space to identify groups of cells exhibiting similar expression behaviors. Similarity is usually defined in terms of the relative positions of the cells in this low-dimensional space: cells that are close together are more likely to be of the same subtype, whilst cells that are far apart are more likely to be of different types.

We speculated that dropout events may distort the relative positions of cells in the low-dimensional subspace potentially leading to misclassification of cell types. To test this, we utilized single-cell data from two recent studies [15, 16] where the cell type identities had been established and could be used as ground truth in a simulation study. We applied PCA and ZIFA to 30 gene subsets of size 500 that were randomly sampled from each data set and projected the data from an initial 500 dimensions to 10 dimensions. We then trained classifiers, using linear and quadratic discriminant analysis (LDA/QDA), and computed the classification error rate of the classifiers. If the cell types are separated well in the latent space, then it would be possible to construct decision boundaries to segregate the classes perfectly and achieve zero classification error on the training data. If cell type classes overlap, it will not be possible to construct classifiers that will separate all cells into their respective groups. The greater the overlap, the greater the rate of misclassification. We treated these misclassification errors as measures of *cell type separability*.

**Fig. 5:**
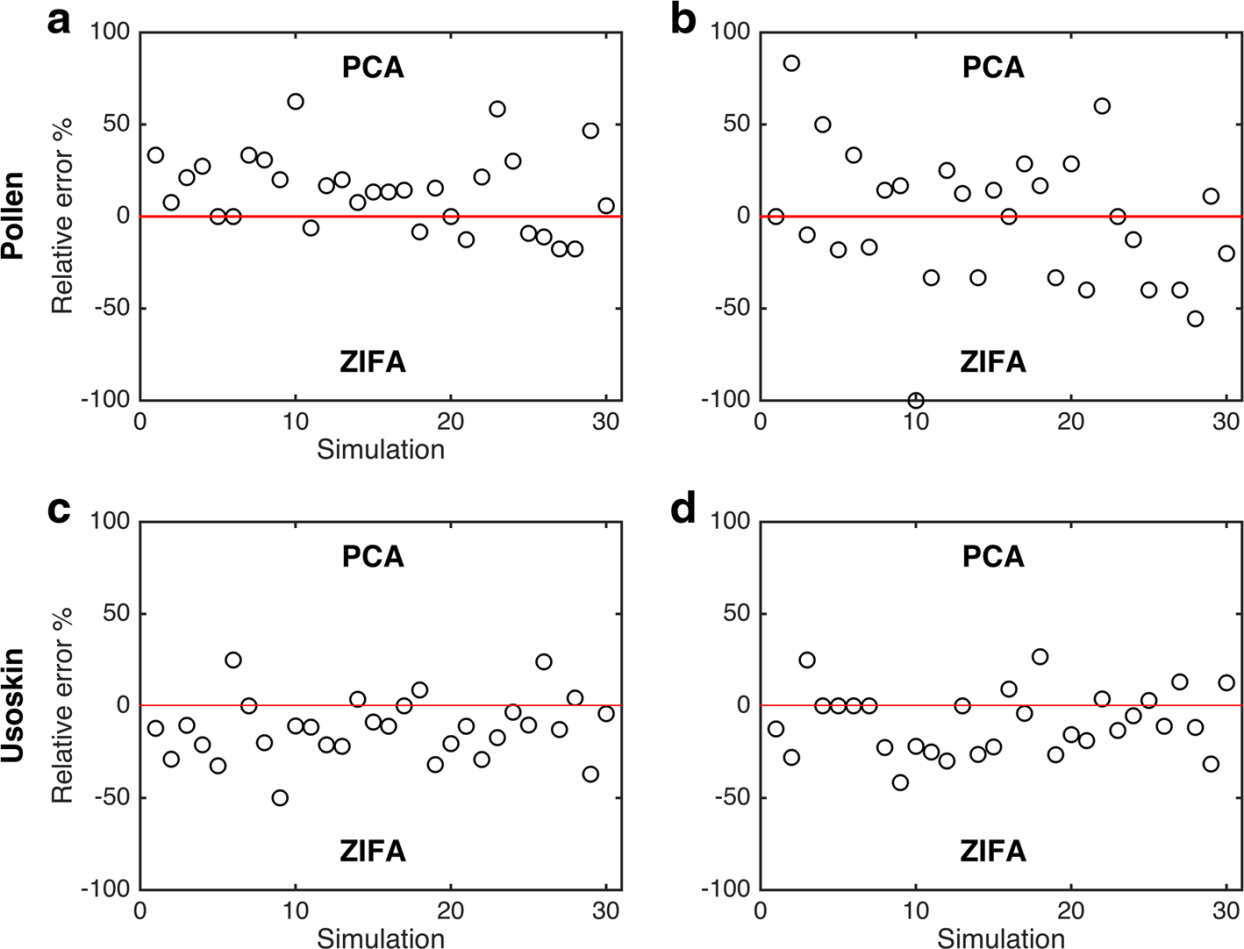
Cell type separability. Plot shows relative cell type misclassification error rates after applying PCA and ZIFA on random subset of 500 genes sampled for the Pollen [15] and Usoskin [16] data sets. Performance was measured based on error rates from (**a**, **c**) linear and (**b**, **d**) quadratic discriminant classifiers. Positive values indicate better performance based on PCA, and negative values for ZIFA

Figure 5 shows that dimensionality reduction using ZIFA led to lower classification error rates than PCA on the Usoskin data [16] indicating that, by taking into account dropout events, ZIFA was able to separate cell types better than PCA. For the Pollen data set, PCA showed better performance than ZIFA when classification error was measured based on an LDA classifier but equal performance when using QDA. It should be noted that overall absolute classification errors for the Pollen data [15] were extremely low (0–2 % using QDA). This is unsurprising as the cell types in this study were derived from a number of unrelated cell lines. Therefore, a comparison of the performance of PCA and ZIFA for this data may not necessarily reflect most experimental conditions. In contrast, the four cell types we considered in the Usoskin data are all neuronal cells.

The previous simulation study was limited because each of the gene subsets had very similar dropout rates that were approximately 50 % and 60 %, respectively, for the Pollen and Usoskin data sets (Additional file 1: Figure S5). To understand better the relationship between the performance of PCA and ZIFA and dropout rate, we used these data sets as a scaffold upon which to construct further simulated data sets. Using simulations allows us to control the rate of dropout events. Our double exponential dropout model was used to introduce dropouts by varying the decay parameter *λ* used in the simulations. The simulation algorithm is detailed in Additional file 1.

**Fig. 6:**
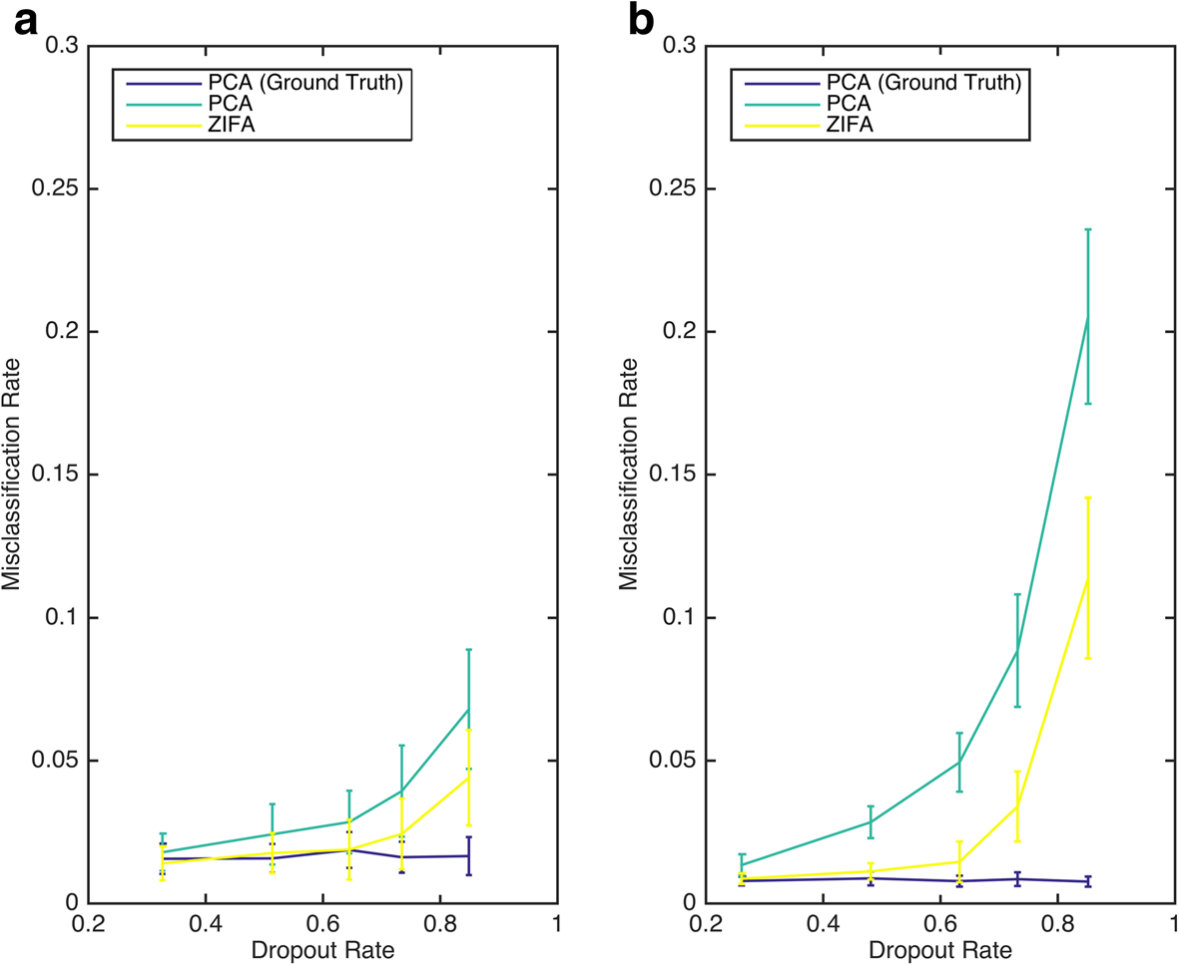
Understanding the relationship between cell type separability and dropout rate. This is a comparison of dimensionality-reduction techniques for cell typing. These plots show cell type misclassification rates (using QDA) as a function of dropout rate for the preprocessing using PCA and ZIFA on simulated data sets based on the (**a**) Pollen [15] and (**b**) Usoskin [16] data sets. The exact PCA results correspond to a ground-truth baseline when PCA is applied to simulated data with no dropout events

Figure 6 shows the relative performance of PCA and ZIFA on the simulated data sets. As the data were simulated, we can also provide a baseline performance from classifiers built from PCA applied to the latent expression measurements with no dropout events (i.e., treating the latent measurements **X** as the observations instead of the zero-inflated observations **Y**). The results show that for low dropout rates, the performance of PCA and ZIFA converges to the baseline. However, at higher dropout rates, ZIFA proves more effective at maintaining cell type separation than PCA for both data sets. We observed from the magnitude of the absolute misclassification errors that separating the neuronal cell types in the Usoskin data is more challenging than with the cell types in the Pollen data set. Classification performance quickly declines as dropout rates increase with the Usoskin data but, even when the average dropout rate was nearly 90 %, it was still possible to achieve less than 10 % misclassification errors with the Pollen data.

In conclusion, the performance gain of ZIFA over PCA for cell type identification problems will heavily depend on the intrinsic separability of the cell subtypes and the dropout rate. Our analysis of the Pollen data suggests there is little to gain from ZIFA over PCA for cell types that are straightforward to separate and would be expected to lie far apart in latent space. However, the Usoskin results suggest there may be greater advantages from modeling dropouts when cellular expression behaviors are more similar and the positions of the cells in latent space are close.

## Discussion

The density of dropout events in scRNA-seq data can render classical dimensionality-reduction algorithms unsuitable and to date it has not been possible to assess the potential ramifications of applying such methods on zero-inflated data. We have modified the PPCA/FA framework to account for dropout to produce a safe method for the dimensionality reduction of single-cell gene expression data that provides robustness against such uncertainties. In the absence of dropout events, the method is essentially equivalent to PPCA/FA, and therefore, software implementations can straightforwardly substitute our approach for existing methods (e.g., Z = PCA(Y, k) to Z = ZIFA(Y, k)). Therefore, users could use ZIFA as a direct substitute with the benefit that it will automatically account for dropouts whereas remedial efforts may be required with standard PCA. Note that our methodology differs from approaches, such as the many variants of robust PCA, that aim to model corrupted observations. ZIFA treats dropouts as real observations, not outliers, whose occurrence properties have been characterized using an empirically informed statistical model.

The inclusion of a zero-inflation model gives ZIFA greater expressive power than standard PPCA/FA but increases the computational complexity. We have developed an approximate inference method for ZIFA and shown that it is possible to handle usefully larger data sets involving thousands of genes and hundreds of samples. Whilst improved approximation methods and parallelization could yield further performance gains, a particularly important factor in determining computational complexity is the selection of the gene set. Potential users should note that ZIFA attempts to *impute* latent expression values for zero measurements. If a gene has a very low frequency of expression and is zero across most cells, this imputation process is unlikely to yield further information and these genes are best removed before analysis to avoid redundant computations.

One of the limitations of ZIFA is that it models strictly zero measurements rather than near-zero values. It has been possible to account for near-zero values in a univariate mixture modeling framework by placing a small-variance distribution around zero rather than a point mass [7, 8]. Achieving the same goal, in a multivariate context, requires further methodological thought and development to produce solutions that are computationally tractable with a large number of dimensions.

Finally, the ZIFA framework lies strictly in the linear transformation framework but non-linear dimensionality-reduction approaches, such as t-SNE [11], have proven to be highly effective in single-cell expression analysis. There are ongoing investigations to determine how zero-inflation can be formally accounted for with such methods. A natural direction would be to incorporate it directly in a non-linear generative approach such as the Gaussian process latent variable model (GP-LVM) [17]. ZIFA is also potentially applicable to other zero-inflated data where there is a negative correlation between the frequency with which a measurement feature is zero and its mean signal magnitude in non-zero samples.

## Additional file

Additional file 1
**Supplementary information.** Supplementary figures and methods. (PDF 1710 kb)

## Abbreviations

EM: Expectation-maximization
FA: Factor analysis
PCA: Principal components analysis
PPCA: Probabilistic principal components analysis
ScRNA-seq: Single-cell RNA expression analysis
ZIFA: Zero-inflated factor analysis

## References

[1] Shapiro E, Biezuner T, Linnarsson S (2013). Single-cell sequencing-based technologies will revolutionize whole-organism science. Nat Rev Genet.

[2] Blainey PC, Quake SR (2014). Dissecting genomic diversity, one cell at a time. Nat Methods.

[3] Buettner F, Natarajan KN, Casale FP, Proserpio V, Scialdone A, Theis FJ (2015). Computational analysis of cell-to-cell heterogeneity in single-cell RNA-sequencing data reveals hidden subpopulations of cells. Nat Biotechnol.

[4] Treutlein B, Brownfield DG, Wu AR, Neff NF, Mantalas GL, Espinoza FH (2014). Reconstructing lineage hierarchies of the distal lung epithelium using single-cell RNA-seq. Nature.

[5] Trapnell C, Cacchiarelli D, Grimsby J, Pokharel P, Li S, Morse M (2014). The dynamics and regulators of cell fate decisions are revealed by pseudotemporal ordering of single cells. Nat Biotechnol.

[6] Patel AP, Tirosh I, Trombetta JJ, Shalek AK, Gillespie SM (2014). Single-cell RNA-seq highlights intratumoral heterogeneity in primary glioblastoma. Science.

[7] Kharchenko PV, Silberstein L, Scadden DT (2014). Bayesian approach to single-cell differential expression analysis. Nat Methods.

[8] Satija R, Farrell JA, Gennert D, Schier AF, Regev A (2015). Spatial reconstruction of single-cell gene expression data. Nat Biotechnol.

[9] Tipping ME, Bishop CM (1999). Probabilistic principal component analysis. J R Stat Soc: Series B (Statistical Methodology).

[10] Islam S, Zeisel A, Joost S, La Manno G, Zajac P, Kasper M (2014). Quantitative single-cell RNA-seq with unique molecular identifiers. Nat Methods.

[11] Van der Maaten L, Hinton G (2008). Visualizing data using t-SNE. J Mach Learn Res.

[12] Tenenbaum JB, De Silva V, Langford JC (2000). A global geometric framework for nonlinear dimensionality reduction. Science.

[13] Kruskal JB (1964). Multidimensional scaling by optimizing goodness of fit to a nonmetric hypothesis. Psychometrika.

[14] Shalek AK, Satija R, Shuga J, Trombetta JJ, Gennert D, Lu D (2014). Single-cell RNA-seq reveals dynamic paracrine control of cellular variation. Nature.

[15] Pollen AA, Nowakowski TJ, Shuga J, Wang X, Leyrat AA, Lui JH (2014). Low-coverage single-cell mRNA sequencing reveals cellular heterogeneity and activated signaling pathways in developing cerebral cortex. Nat Biotechnol.

[16] Usoskin D, Furlan A, Islam S, Abdo H, Lönnerberg P, Lou D (2015). Unbiased classification of sensory neuron types by large-scale single-cell RNA sequencing. Nat Neurosci.

[17] Lawrence N (2005). Probabilistic non-linear principal component analysis with Gaussian process latent variable models. J Mach Learn Res.

