# Supplementary material for: ZIFA: Dimensionality reduction for zero-inflated single-cell gene expression analysis
Source: Genome Biol. 2015 Nov 2;16:241. doi: 10.1186/s13059-015-0805-z (PMC4630968; doi:10.1186/s13059-015-0805-z)
Supplement: Additional file 1 — Supplementary information. Supplementary figures and methods. (PDF 1710 kb) [file 13059_2015_805_MOESM1_ESM.pdf]

Supplementary Information

ZIFA: Dimensionality reduction for zero-inflated single-cell gene expression analysis

Emma Pierson*[1] and Christopher Yau†[1,2]

[1]Department of Statistics, University of Oxford, 1 South Parks Road, Oxford, OX1 3TG, United Kingdom.
[2]Wellcome Trust Centre for Human Genetics, University of Oxford, Roosevelt Drive, Oxford, OX3 7BN, United Kingdom.

October 13, 2015

Contents

1 Supplementary Methods **3**
- 1.1 Setup . . . 3
- 1.2 Statistical Inference . . . 3
  - 1.2.1 Complete data log-likelihood . . . 4
  - 1.2.2 E-step . . . 4
  - 1.2.3 M-step . . . 6
- 1.3 Fast linear algebra implementations . . . 7
- 1.4 Block ZIFA approximation . . . 8
- 1.5 Initialization of parameters . . . 9

2 Single cell data simulation **9**
- 2.1 Procedure . . . 9
- 2.2 Preprocessing and fitting . . . 10

3 Supplementary Figures **11**

List of Figures

1 Single cell dataset [3] using unique molecular identifiers (UMIs). The relationship between zero expression occurrence and expression level fits the proposed model well. 11

*epierson@cs.stanford.edu
†cyau@well.ox.ac.uk

2 Correlation between parameter estimates from the exact and block-based approximate EM algorithms. Data was simulated according to the generative model with parameters $\lambda = 0.05$, $A_{ij} \sim N(0,1)$, $\mu_j \sim N(6,1)$ and $\sigma_j^2 \sim U(0,1)$ with $n = 200$ and $p = 500$. A block size of 100 used for the approximate algorithm. Compute time taken for 5 EM iterations was 28 and 117 seconds for the block-based and exact EM algorithms respectively. 12
3 Performance of dimensionality reduction methods on simulated data using (A) linear decay $1 - \lambda\mu_0$ and (B) missing at random $1 - \lambda$ zero-inflation models. 13
4 Performance of dimensionality reduction methods on simulated data using a squared-error metric, with lower scores denoting superior performance: (A) double-exponential decay, (B) linear decay $1 - \lambda\mu_0$ and (C) missing at random $1 - \lambda$ zero-inflation models. 14
5 Distribution of average dropout rates over the random 500-gene subsets sampled from the Pollen [1] and Usoskin [2] data sets. 15

1 Supplementary Methods

In this section we provide extensive detail regarding the mathematical set-up and derivation of the proposed zero-inflated factor analysis model.

1.1 Setup

Let N be the number of samples, D be the number of genes, and K be the desired number of latent dimensions. The data is given by a high-dimensional $N \times D$ data matrix $\mathbf{Y} = [\mathbf{y}_1, \ldots, \mathbf{y}_N]$, where y_{ij} is the level of expression (log read count) of the j-th gene in the i-th sample. The data is assumed to be generated from a projection of a latent low-dimensional $N \times K$ matrix $\mathbf{Z} = [\mathbf{z}_1, \ldots, \mathbf{z}_N]$ ($K \ll D$). In all derivations below, we use use $i = 1, ..., N$ to index over samples (cells), $j = 1, ..., D$ to index over genes, and $k = 1, ..., K$ to index over latent dimensions. Each sample $\mathbf{y}_i$ is drawn independently:

$$\mathbf{z}_i \sim \text{Normal}(0, \mathbf{I}), \tag{1}$$

$$\mathbf{x}_i | \mathbf{z}_i \sim \text{Normal}(\mathbf{A}\mathbf{z}_i + \boldsymbol{\mu}, \mathbf{W}), \tag{2}$$

$$h_{ij} | x_{ij} \sim \text{Bernoulli}(\exp(-\lambda x_{ij}^2)), \tag{3}$$

$$y_{ij} = \begin{cases} x_{ij}, & \text{if } h_{ij} = 0, \\ 0, & \text{if } h_{ij} = 1, \end{cases} \tag{4}$$

where $\mathbf{I}$ denotes the $K \times K$ identity matrix, $\mathbf{A}$ denotes a $D \times K$ factor loadings matrix, $\mathbf{H}$ is a $D \times N$ masking matrix, $\mathbf{W} = \text{diag}(\sigma_1^2, \ldots, \sigma_D^2)$ is a $D \times D$ diagonal matrix, $\boldsymbol{\mu}$ is a $D \times 1$ mean vector, and λ is the exponential decay parameter in the zero-inflation model. Note that λ is shared across genes which reduces the number of parameters to be estimated and captures the fact that technical noise should have similar effects across genes.

1.2 Statistical Inference

Given an observed single cell gene expression matrix $\mathbf{Y}$ we wish to identify model parameters $\Theta = (A, \sigma^2, \mu, \lambda)$ that maximize the likelihood $p(\mathbf{Y}|\theta)$. We do this using the expectation-maximization (EM) algorithm. We summarize the algorithm in the box below and then describe the algebraic details:

Algorithm 1: EM for Zero-Inflated Dimensionality Reduction

```
1 initialize model parameters A, μ, σ², λ;
2 while parameters not converged do
3 |   E-step: given A, μ, σ², λ, compute p(Z, X_0|Y) and E[Z], E[ZZ^T], E[X_0], E[X_0^2], E[X_0 Z]
  |   where X_0 is the subset of X where corresponding elements in Y are zero;
4 |   M-step: compute analytic updates for A, μ, σ² and optimize λ numerically;
5 end
```

We denote the value of the parameters at the n-th iteration, Θ_n, as the value that maximizes the expected value of the complete log likelihood $p(\mathbf{Z}, \mathbf{X}, \mathbf{H}, \mathbf{Y})$ under the conditional distribution over the latent variables given the observed data and the parameters at the last iteration. Computing

the value of the parameters at each iteration requires two steps: the expectation step (E-step) and the maximization step (M-step). In the E-step, we derive an expression for the complete log likelihood $p(\mathbf{Z}, \mathbf{X}, \mathbf{H}, \mathbf{Y}|\Theta_n)$ and compute all necessary expectations under the distribution $p(\mathbf{Z}, \mathbf{X}, \mathbf{H}|\mathbf{Y}, \Theta_{n-1})$. In the M-step, we maximize the expected value of the complete log likelihood with respect to Θ_n.

1.2.1 Complete data log-likelihood

The complete data likelihood is given by:

$$p(\mathbf{Z}, \mathbf{X}, \mathbf{H}, \mathbf{Y}|\Theta) = \prod_{i=1}^{N} P(\mathbf{z}_i)p(\mathbf{x}_i|\mathbf{z}_i)p(\mathbf{h}_i|\mathbf{x}_i)p(\mathbf{y}_i|\mathbf{h}_i, \mathbf{x}_i), \tag{5}$$

$$= \prod_{i=1}^{N} \left[p(\mathbf{z}_i) \prod_{j=1}^{p} p(x_{ij}|\mathbf{z}_i)p(h_{ij}|x_{ij})p(y_{ij}|x_{ij}, h_{ij}) \right] \tag{6}$$

There are two different cases to consider: $y_{ij} \neq 0, h_{ij} = 0$, and $y_{ij} = 0, h_{ij} = 1$. Note the case where $y_{ij} = 0, h_{ij} = 0$ has probability zero because for $y_{ij} = 0$ when $h_{ij} = 0$ we must have $x_{ij} = 0$, which means that $h_{ij} = 1$ with probability $\exp(-\lambda x_{ij}^2) = 1$. The case where $y_{ij} \neq 0, h_{ij} = 1$ has probability zero because $h_{ij} = 1 \rightarrow y_{ij} = 0$. Thus,

$$p(\mathbf{Z}, \mathbf{X}, \mathbf{H}, \mathbf{Y}|\Theta) = \prod_{i=1}^{N} \left[p(\mathbf{z}_i) \prod_{j=1}^{p} p(x_{ij}|\mathbf{z}_i)p(h_{ij}|x_{ij})p(y_{ij}|x_{ij}, h_{ij}) \right], \tag{7}$$

$$= \prod_{i=1}^{N} \left[p(\mathbf{z}_i) \prod_{j:y_{ij}=0} p(x_{ij}|\mathbf{z}_i)p(h_{ij} = 1|x_{ij}) \prod_{j:y_{ij}\neq 0} p(x_{ij} = y_{ij}|\mathbf{z}_i)p(h_{ij} = 0|x_{ij}) \right] \tag{8}$$

Taking the log of this and substituting in the expressions from the generative model yields the log likelihood ($\tilde{\mathbf{x}}_i = \mathbf{A}\mathbf{z}_i + \boldsymbol{\mu}$):

$$\begin{aligned} \ln p(\mathbf{Z}, \mathbf{X}, \mathbf{H}, \mathbf{Y}|\Theta) \propto & -\frac{1}{2} \sum_{i=1}^{N} \mathbf{z}_i^T \mathbf{z}_i \\ & + \sum_{i=1}^{N} \left[\sum_{j:y_{ij}=0} \left\{ -\frac{(x_{ij} - \tilde{x}_{ij})^2}{2\sigma_j^2} - \frac{1}{2} \log \sigma_j^2 - \lambda x_{ij}^2 \right\} \right] \\ & + \sum_{i=1}^{N} \left[\sum_{j:y_{ij}=0} \left\{ -\frac{(y_{ij} - \tilde{x}_{ij})^2}{2\sigma_j^2} - \frac{1}{2} \log \sigma_j^2 + \ln(1 - \exp(-\lambda y_{ij}^2)) \right\} \right] \end{aligned}$$

1.2.2 E-step

The E-step requires us to compute expectations for z_{ik}, $z_{ik}^2, x_{ij}, x_{ij}^2, z_{ij}z_{ik}$, and $x_{ij}z_{ik}$ under the conditional distribution $p(\mathbf{z}_i, \mathbf{x}_i|\mathbf{y}_i, \Theta)$. We use $\mathbf{y}_{i+}$ to denote the elements of $\mathbf{y}_i$ that are non-zero and $\mathbf{y}_{i0}$ to denote the elements of $\mathbf{y}_i$ that are zero (similarly for $\mathbf{x}_i$). We are only interested in the distributions over $\mathbf{x}_{i0}$, since $\mathbf{x}_{i+}$ is effectively observed through $\mathbf{y}_i$; thus, we need the distribution

$p(\mathbf{z}_i, \mathbf{x}_{i0}|\mathbf{y}_i)$:

$$
\begin{aligned}
p(\mathbf{z}_i, \mathbf{x}_{i0}|\mathbf{y}_i) &= p(\mathbf{z}_i, \mathbf{x}_{i0}|\mathbf{y}_{i0}, \mathbf{y}_{i+}), && (9)\\
&\propto p(\mathbf{y}_{i0}, \mathbf{z}_i, \mathbf{x}_{i0}|\mathbf{y}_{i+}), && (10)\\
&= p(\mathbf{z}_i, \mathbf{x}_{i0}|\mathbf{y}_{i+})p(\mathbf{y}_{i0}|\mathbf{x}_{i0}) && (11)
\end{aligned}
$$

The second term is easy to compute since we have $p(\mathbf{y}_{i0}|\mathbf{x}_{i0}) = \exp(-\lambda \mathbf{x}_{i0}^2)$. Thus we can compute $p(\mathbf{z}_i, \mathbf{x}_{i0}|\mathbf{y}_{i+})$ as follows. We know the prior distribution $p(\mathbf{z}_i, \mathbf{x}_i)$ is a multivariate normal with prior mean $\boldsymbol{\mu}^{(p)}$ given by the vector of length $(K + D)$

$$
\boldsymbol{\mu}^{(p)} = \begin{pmatrix} 0 \\ \boldsymbol{\mu} \end{pmatrix} \tag{12}
$$

where the upper 0 denotes the first K entries and the μ denotes the last D entries. The prior covariance $\Sigma^{(p)}$ is given by the $(K + D) \times (K + D)$ matrix

$$
\boldsymbol{\Sigma}^{(p)} = \begin{pmatrix} I & \mathbf{A}^T \\ \mathbf{A} & \mathbf{A}\mathbf{A}^T + \mathbf{W} \end{pmatrix} \tag{13}
$$

In order to obtain the distribution of $\mathbf{z}_i, \mathbf{x}_{i0}$ conditional on $\mathbf{x}_{i+} = \mathbf{y}_{i+}$ we use the fact that the conditional distribution of a multivariate normal is also a multivariate normal with mean $\boldsymbol{\mu}_c$ and covariance $\boldsymbol{\Sigma}_c$ given by:

$$
\begin{aligned}
\boldsymbol{\mu}_c &= \boldsymbol{\mu}_0^{(p)} + \boldsymbol{\Sigma}_{0+}^{(p)}(\boldsymbol{\Sigma}_{++}^{(p)})^{-1}(\mathbf{y}_{i+} - \boldsymbol{\mu}_{+}^{(p)}), && (14)\\
\boldsymbol{\Sigma}_c &= \boldsymbol{\Sigma}_{00}^{(p)} - \boldsymbol{\Sigma}_{0+}^{(p)}(\boldsymbol{\Sigma}_{++}^{(p)})^{-1}\boldsymbol{\Sigma}_{+0}^{(p)} && (15)
\end{aligned}
$$

where $\boldsymbol{\mu}_{+}^{(p)}$ denotes the entries of $\boldsymbol{\mu}^{(p)}$ corresponding to $\mathbf{x}_{i+}$, and $\boldsymbol{\mu}_0^{(p)}$ denotes the other entries; partition $\boldsymbol{\Sigma}^{(p)}$ similarly, e.g. $\boldsymbol{\Sigma}_{0+}^{(p)}$ is the sub-matrix of $\boldsymbol{\Sigma}^{(p)}$ formed from the rows whose indices are given by the indices where $\mathbf{y}_i$ is zero and columns whose indices are given by the indices where $\mathbf{y}_i$ is non-zero.

Thus, $p(\mathbf{z}_i, \mathbf{x}_{i0}|\mathbf{y}_i)$ is also is a multivariate normal distribution with mean

$$
(\boldsymbol{\Sigma}_c^{-1} + 2\lambda \mathbf{I}_x)^{-1}\boldsymbol{\Sigma}_c^{-1}\boldsymbol{\mu}_c \tag{16}
$$

and covariance

$$
(\boldsymbol{\Sigma}_c^{-1} + 2\lambda \mathbf{I}_x)^{-1} \tag{17}
$$

where $\mathbf{I}_x$ is the diagonal matrix with ones on diagonal elements corresponding to entries of $\mathbf{x}_{i0}$ and zeros everywhere else. Although these formulae involve inverses of high-dimensional matrices, the expressions can be simplified such that no high-dimensional inversions are required; we describe how to do this in a later section.

1.2.3 M-step

In the M-step we first update $\mathbf{A}$ and $\boldsymbol{\mu}$; then we update $\mathbf{W}$, whose value depends on $\mathbf{A}$ and $\boldsymbol{\mu}$; then we update λ. All updates are analytic except for λ which we optimize numerically. We want to maximize the expected value of the complete data log-likelihood with respect to the parameters. Collecting the terms that contain μ_j and setting $\frac{d}{d\mu_j} = 0$ yields

$$\mu_j = \frac{1}{N}\Big[\sum_{i:y_{ij}=0}\Big(E[x_{ij}] - \sum_{k=1}^{K} a_{jk}E[z_{ik}]\Big) + \sum_{i:y_{ij}>0}\Big(y_{ij} - \sum_{k=1}^{K} a_{jk}E[z_{ik}]\Big)\Big] \tag{18}$$

Doing the same for a_{jk} yields

$$a_{jk} = \frac{1}{\sum_{i=1}^{N} E[z_{ik}^2]}\Big[\sum_{i:y_{ij}=0}\Big(E[x_{ij}z_{ik}] - \mu_j E[z_{ik}] - \sum_{k'\neq k} a_{jk'}E[z_{ik}z_{ik'}]\Big) + \sum_{i:y_{ij}>0}\Big(Y_{ij}E[z_{ik}] - \mu_j E[z_{ik}] - \sum_{k'\neq k} a_{jk'}E[z_{ik}z_{ik'}]\Big)\Big] \tag{19}$$

The optimal value for μ_j depends on $a_{j1}, a_{j2}, ..., a_{jK}$ and vice versa. We can express these constraints as a matrix equation for each j and solve them for each j independently. We have $B_j u_j - c_j = 0$, where B_j is a $(D+1) \times (D+1)$ matrix and u_j and c_j are vectors of length $(D+1)$:

$$u_j = \begin{pmatrix} a_{j1} \\ a_{j2} \\ \vdots \\ a_{jD} \\ \mu_j \end{pmatrix} \tag{20}$$

$$B_j = \begin{pmatrix} 1 & \frac{\sum_i E[z_{i1}z_{i2}]}{\sum_i E[z_{i1}^2]} & \dots & \frac{\sum_i E[z_{i1}]}{\sum_i E[z_{i1}^2]} \\ \frac{\sum_i E[z_{i2}z_{i1}]}{\sum_i E[z_{i2}^2]} & 1 & \dots & \frac{\sum_i E[z_{i2}]}{\sum_i E[z_{i2}^2]} \\ \vdots & \vdots & \ddots & \vdots \\ \frac{1}{N}\sum_i E[z_{i1}] & \frac{1}{n}\sum_i E[z_{i2}] & \dots & 1 \end{pmatrix} \tag{21}$$

$$c_j = \begin{pmatrix} \frac{\sum_{i:y_{ij}=0} E[x_{ij}z_{i1}] + \sum_{i:y_{ij}>0} y_{ij}E[z_{i1}]}{\sum_i E[z_{i1}^2]} \\ \frac{\sum_{i:y_{ij}=0} E[x_{ij}z_{i2}] + \sum_{i:y_{ij}>0} y_{ij}E[z_{i2}]}{\sum_i E[z_{i2}^2]} \\ \vdots \\ \frac{1}{N}\Big(\sum_{i:y_{ij}=0} E[x_{ij}] + \sum_{i:y_{ij}>0} y_{ij}\Big) \end{pmatrix} \tag{22}$$

Having solved for μ_j and $a_{j1}, a_{j2}, ..., a_{jK}$, we use the updated values to update σ_j^2. Defining $m_{ij} = \sum_{k=1}^{K} a_{jk} z_{ik} + \mu_j$, we have

$$\sigma_j^2 = \frac{1}{N}\Big(\sum_{i:y_{ij}=0}(E[x_{ij}^2] - 2E[x_{ij}m_{ij}] + E[m_{ij}^2]) + \sum_{i:y_{ij}>0}(y_{ij}^2 - 2y_{ij}E[m_{ij}] + E[m_{ij}^2])\Big) \tag{23}$$

This expression is very similar to the expression for the variance in factor analysis, but with y_{ij} replaced with the expected value of x_{ij} for zero values of y_{ij}. This makes sense because when y_{ij} is zero, x_{ij} is unobserved, but x_{ij} is otherwise equal to y_{ij}. We note that constraining all σ_j^2 to be equal yields zero-inflated probabilistic principal components analysis rather than zero-inflated factor analysis: we have

$$\sigma^2 = \frac{1}{ND}\sum_j\Big(\sum_{i:y_{ij}=0}(E[x_{ij}^2] - 2E[x_{ij}m_{ij}] + E[m_{ij}^2]) + \sum_{i:y_{ij}>0}(y_{ij}^2 - 2y_{ij}E[m_{ij}] + E[m_{ij}^2])\Big) \tag{24}$$

We then optimize λ numerically, and the M-step is complete.

1.3 Fast linear algebra implementations

In the E-step, our goal is to compute the mean and covariance for the distribution $p(Z_i, X_{i0}|Y_i)$. These are given by

$$(\Sigma_c^{-1} + 2\lambda I_x)^{-1}\Sigma_c^{-1}\mu_c \tag{25}$$

$$(\Sigma_c^{-1} + 2\lambda I_x)^{-1} \tag{26}$$

These computations are potentially expensive because they involve inverting $D \times D$ matrices, and matrix inversion has cubic complexity. We show that it is possible to evaluate these expressions without computing the inverse of any $D \times D$ matrix, which greatly decreases the runtime of our algorithm.

We first simplify $(\mathbf{\Sigma}_c^{-1} + 2\lambda\mathbf{I}_x)^{-1}$ using the Kailath variant of the Woodbury identity which yields

$$(\mathbf{\Sigma}_c^{-1} + 2\lambda\mathbf{I}_x)^{-1} = \mathbf{\Sigma}_c - 2\lambda\mathbf{\Sigma}_c(\mathbf{I} + 2\lambda\mathbf{I}_x\mathbf{\Sigma}_c)^{-1}\mathbf{I}_x\mathbf{\Sigma}_c \tag{27}$$

Thus, we can write the mean and covariance of $p(Z_i, X_{i0}|Y_i)$ as

$$(\mathbf{I} - 2\lambda\mathbf{\Sigma}_c(\mathbf{I} + 2\lambda\mathbf{I}_x\mathbf{\Sigma}_c)^{-1}\mathbf{I}_x)\boldsymbol{\mu}_c \tag{28}$$

$$\mathbf{\Sigma}_c - 2\lambda\mathbf{\Sigma}_c(I + 2\lambda\mathbf{I}_x\mathbf{\Sigma}_c)^{-1}\mathbf{I}_x\mathbf{\Sigma}_c \tag{29}$$

These expressions contains the inverse of $(\mathbf{I} + 2\lambda\mathbf{I}_x\mathbf{\Sigma}_c)$. We use the block matrix inversion formula to compute this, which yields

$$(\mathbf{I} + 2\lambda\mathbf{I}_x\mathbf{\Sigma}_c)^{-1} = \begin{pmatrix} \mathbf{I} & 0 \\ -(\mathbf{I} + 2\lambda\mathbf{\Sigma}_{xx})^{-1}2\lambda\mathbf{\Sigma}_{xz} & (\mathbf{I} + 2\lambda\mathbf{\Sigma}_{xx})^{-1} \end{pmatrix} \tag{30}$$

where $\mathbf{\Sigma}_{xx}, \mathbf{\Sigma}_{xz}$ correspond to the sub matrices of $\mathbf{\Sigma}_c$ corresponding to the x and z indices. Thus,

we need to compute the inverse of $\mathbf{I}+2\lambda\mathbf{\Sigma}_{xx}$. Given the formula for $\mathbf{\Sigma}_c$, we have

$$(\mathbf{I}+2\lambda\mathbf{\Sigma}_{xx}) = \mathbf{I}+2\lambda(\mathbf{\Sigma}_{00}^{(p)} - \mathbf{\Sigma}_{0+}^{(p)}\mathbf{\Sigma}^{(p)}{}_{++}^{-1}\mathbf{\Sigma}_{+0}^{(p)}) \tag{31}$$

$$= 2\lambda(\mathbf{\Sigma}_{00}' - \mathbf{\Sigma}_{0+}^{(p)}\mathbf{\Sigma}^{(p)}{}_{++}^{-1}\mathbf{\Sigma}_{+0}^{(p)}) \tag{32}$$

where the 0 and + subscripts denote the indices of $\mathbf{x}_i$ which correspond to zero and non-zero entries in $\mathbf{y}_i$, respectively, and $\mathbf{\Sigma}_{00}' = \mathbf{\Sigma}_{00}^{(p)} + \frac{1}{2\lambda}\mathbf{I}$.

We can invert this expression by applying another form of the Woodbury formula:

$$(E+CBC^T)^{-1} = E^{-1} - E^{-1}C(B^{-1}+C^TE^{-1}C)^{-1}C^TE^{-1} \tag{33}$$

with $E = \mathbf{\Sigma}_{00}', B = -\mathbf{\Sigma}_{++}^{-1}, C = \mathbf{\Sigma}_{0+}$. This yields

$$(\mathbf{\Sigma}_{00}' - \mathbf{\Sigma}_{0+}^{(p)}\mathbf{\Sigma}^{(p)}{}_{++}^{-1}\Sigma^{(p)}{}_{+0})^{-1} = \mathbf{\Sigma}'^{-1}_{00} - \Sigma'^{-1}_{00}\Sigma_{0+}^{(p)}(-\Sigma_{++}^{(p)} + \mathbf{\Sigma}_{+0}^{(p)}\Sigma'^{-1}_{00}\mathbf{\Sigma}^{(p)}{}_{0+})^{-1}\mathbf{\Sigma}_{+0}^{(p)}\mathbf{\Sigma}'^{-1}_{00} \tag{34}$$

$$= \mathbf{\Sigma}'^{-1}_{00} - \mathbf{\Sigma}'^{-1}_{00}\mathbf{\Sigma}_{0+}^{(p)}(-\mathbf{\Sigma}_{++}^{(p)} + \mathbf{A}_+(\mathbf{A}_0^T\mathbf{\Sigma}'^{-1}_{00}\mathbf{A}_0)\mathbf{A}_+^T)^{-1}\mathbf{\Sigma}_{+0}^{(p)}\mathbf{\Sigma}'^{-1}_{00} \tag{35}$$

where $\mathbf{A}_0$ and $\mathbf{A}_+$ denote the rows of $\mathbf{A}$ corresponding to zero and non-zero entries of $\mathbf{y}_i$, respectively.

We can compute the inverse $(-\mathbf{\Sigma}_{++}^{(p)} + \mathbf{A}_+(\mathbf{A}_0^T\mathbf{\Sigma}'^{-1}_{00}\mathbf{A}_0)\mathbf{A}_+^T)^{-1}$ by applying the Woodbury formula with $E = -\mathbf{\Sigma}_{++}^{(p)}$, $B = \mathbf{A}_0^T\mathbf{\Sigma}'^{-1}_{00}\mathbf{A}_0$, $C = \mathbf{A}_+$. Note that B will be $K \times K$, making it easy to invert. We can invert $\mathbf{\Sigma}_{00}'$ and all $D \times D$ matrices with the form $AA^T + E$ (where A is skinny and E is diagonal) by noting that

$$(AA^T+E)^{-1} = E^{-1} - E^{-1}A(I+A^TE^{-1}A)^{-1}A^TE^{-1} \tag{36}$$

which will be much faster because the inverse $(I + A^TE^{-1}A)$ is $K \times K$ rather than $D \times D$. These simplifications allow us to avoid computing any large inverses, making the most expensive step in the algorithm the multiplications of $D \times D$ matrices. As a final computational speed-up, we note that in many cases these multiplications can be avoided because the $D \times D$ matrices are products of two $D \times K$ matrices, producing large decreases in runtime if an optimal multiplication order is used. Because computations for each sample are independent, this algorithm is easily parallelizable, and could be run on a cluster if greater speed-ups are desired.

1.4 Block ZIFA approximation

In the expectation step, we require computations that use the full conditional distribution $p(\mathbf{z}_i, \mathbf{x}_{i0}|\mathbf{y}_i)$. These computations can be unwieldy for whole transcriptome data due to the need to condition on all genes in the data with non-zero measurement so that we can define the conditional probability over the latent variable $\mathbf{z}_i$ and (zero) measurements $\mathbf{x}_0$. In order to speed up computations for

large datasets, we can approximate the full conditional distribution with the following:

$$\begin{aligned}
p(\mathbf{z}, \mathbf{x}_0, \mathbf{y}) &= p(\mathbf{z}|\mathbf{x}_0, \mathbf{y})p(\mathbf{x}_0, \mathbf{y}), \\
&\approx \sum_{m=1}^{M} \tilde{p}(\mathbf{z}, \mathbf{x}_0, \mathbf{y}, m), \\
&= \sum_{m=1}^{M} \tilde{p}(\mathbf{z}|\mathbf{x}_{0,S_m}, \mathbf{y}_{S_m}, m)\tilde{p}(\mathbf{x}_{0,S_m}, \mathbf{y}_{S_m}|m)p(m),
\end{aligned}$$

where for notational convenience, we drop the subscript i indicating the cell index and conditional dependence on other parameters. This approximation partitions the p genes into M disjoint blocks or subsets $\{S_1, S_2, \ldots, S_M\}$ such that $\bigcup_{m=1}^{M} S_m = \{1, \ldots, p\}$. and approximates the full joint distribution as a uniform mixture ($p(m) = 1/M$) where the latent variable m indicates the subset under consideration.

This approximation modifies the E-step so that expectations for each element of the latent measurements $\mathbf{x}_0$ depends on a much smaller subset of the observations $\mathbf{y}$. Furthermore, as each block of data gives rise to a different prediction for the latent state $\mathbf{z}$, the mixture model allows us to effectively average over the possibilities raised by each subset. This block approximation decreases the runtime from quadratic in the total number of genes to quadratic in the block size. We have found that block sizes $p/M \approx 500$ gives good performance. Note that the approximation allows for trivial parallelization over blocks and cells which would boost computation speed if multiple processing cores are available.

We do not explore the details here but future analysis would involve an examination of the asymptotic properties of the approximation when averaging over random subsets/blocks of the data.

1.5 Initialization of parameters

We initialize A, μ, and W using standard factor analysis: we fit a factor analysis model to Y, and set A, μ, and W to the model parameters. To initialize λ, for each gene j, we compute the mean of non-zero samples μ and the fraction of samples that are zero, p_0; we then fit a decaying squared exponential curve to the pairs (μ, p_0), and set λ to the fitted exponential parameter.

2 Single cell data simulation

This section describes the simulation procedure used to generate simulated data for comparison of ZIFA, PPCA and FA.

2.1 Procedure

Let $\mathbf{Y}$ be a $p \times n$ observed gene expression matrix for p genes and n cells and let $\mathbf{L}$ denote a $n \times 1$ vector of corresponding cell type labels:

1. Perform a PCA on $\mathbf{Y}$ to obtain a $p \times D$ loadings matrix $\mathbf{A}$, $D \times n$ scores matrix $\mathbf{Z}$ and $p \times 1$ centering vector $\boldsymbol{\mu}$:
$$(\mathbf{A}, \mathbf{Z}, \boldsymbol{\mu}) \Leftarrow \texttt{PCA}(\mathbf{Y}, D)$$

2. Perform quadratic discriminant analysis (QDA) on the subspace spanned by the top d principal components and compute the misclassification error e:

$$e(d) \Leftarrow \texttt{QDA}(\mathbf{Z}_d, \mathbf{L})$$

Find d such that $e(d)$ is below 10%.

3. Generate a simulated latent measurement set:

$$\mathbf{X}' \Leftarrow \mathbf{A}_d \mathbf{Z}_d + \boldsymbol{\mu} + \boldsymbol{\epsilon}$$

where $\boldsymbol{\epsilon}$ is a multivariate Gaussian noise vector with zero mean and diagonal covariance matrix where the diagonal elements are randomly drawn from Uniform$(0, 1)$.

4. Introduce drop out events according to the double-exponential model for a given λ:

$$Y'_{ij} = \begin{cases} X'_{ij}, & \text{if } p_{ij} > \exp(-\lambda (X'_{ij})^2), \\ 0, & \text{otherwise.} \end{cases}$$

where $p_{ij} \sim$ Uniform$(0, 1)$.

2.2 Preprocessing and fitting

The Pollen [1] and Usoskin [2] datasets were pre-filtered to exclude genes where more than 90% of cells had zero measurements. For the sensory neural cells from Usoskin data set, we excluded the non-neuronal cells and only used the major four cellular classes ($n = 622$). For the Pollen data, we used only single cells with greater than 500,000 reads ($n = 249$).

We found that $d = 5$ gave good classification results for both data sets (Supplementary Figure 6).

References

1. Pollen, A. A. *et al.* Low-coverage single-cell mRNA sequencing reveals cellular heterogeneity and activated signaling pathways in developing cerebral cortex. *Nature Biotechnology* (2014).
2. Usoskin, D. *et al.* Unbiased classification of sensory neuron types by large-scale single-cell RNA sequencing. *Nature neuroscience* **18,** 145–153 (2015).
3. Islam, S. *et al.* Quantitative single-cell RNA-seq with unique molecular identifiers. *Nature methods* **11,** 163–166 (2014).

3 Supplementary Figures

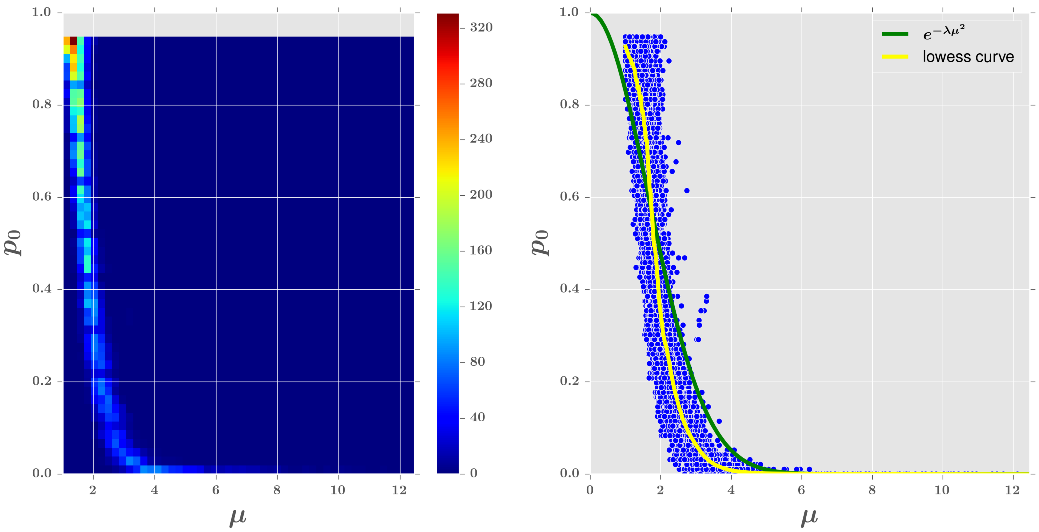

Figure 1: Single cell dataset [3] using unique molecular identifiers (UMIs). The relationship between zero expression occurrence and expression level fits the proposed model well.

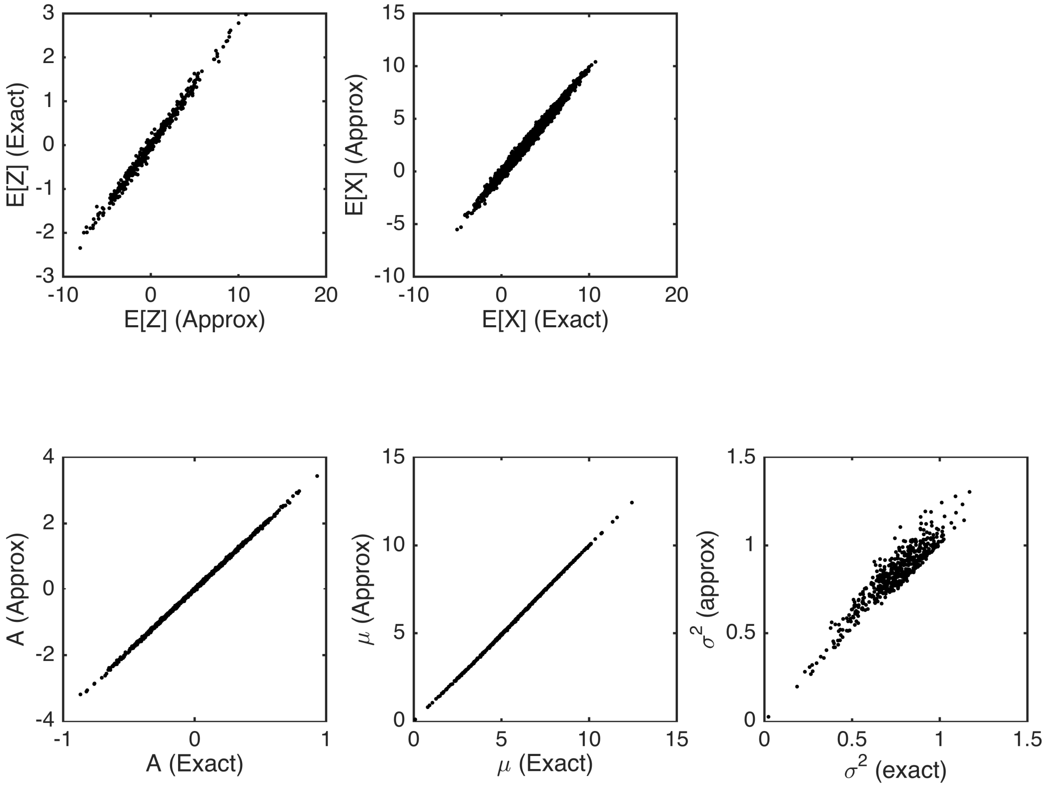

Figure 2: Correlation between parameter estimates from the exact and block-based approximate EM algorithms. Data was simulated according to the generative model with parameters $\lambda = 0.05$, $A_{ij} \sim N(0, 1)$, $\mu_j \sim N(6, 1)$ and $\sigma_j^2 \sim U(0, 1)$ with $n = 200$ and $p = 500$. A block size of 100 used for the approximate algorithm. Compute time taken for 5 EM iterations was 28 and 117 seconds for the block-based and exact EM algorithms respectively.

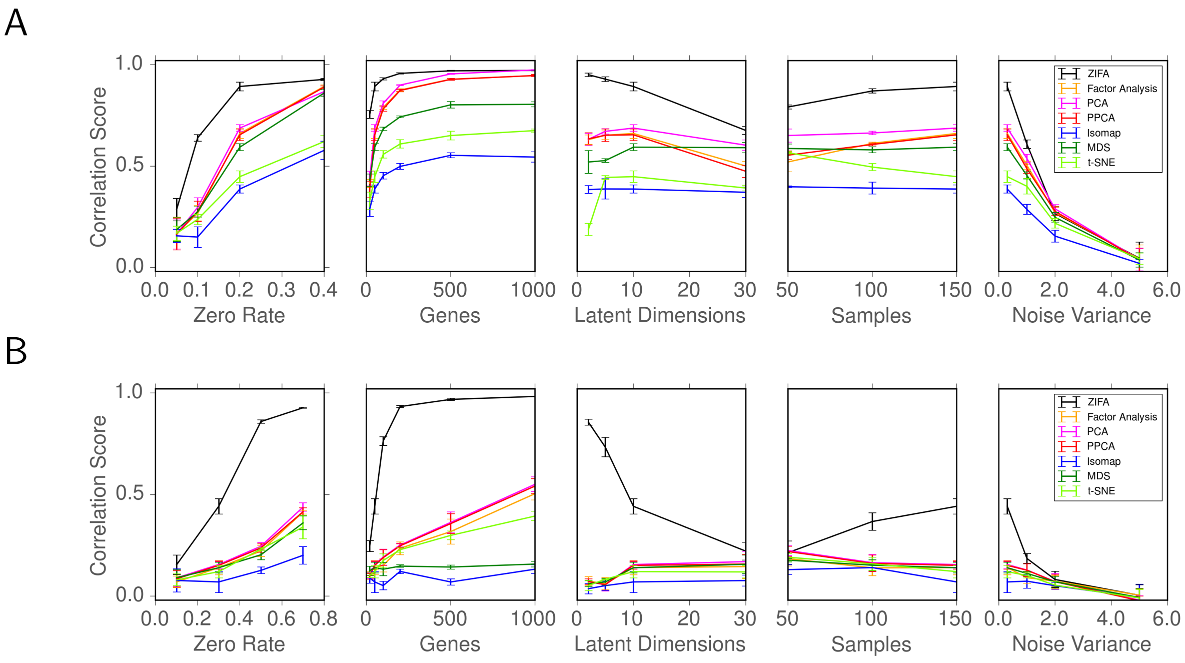

Figure 3: Performance of dimensionality reduction methods on simulated data using (A) linear decay $1 - \lambda\mu_0$ and (B) missing at random $1 - \lambda$ zero-inflation models.

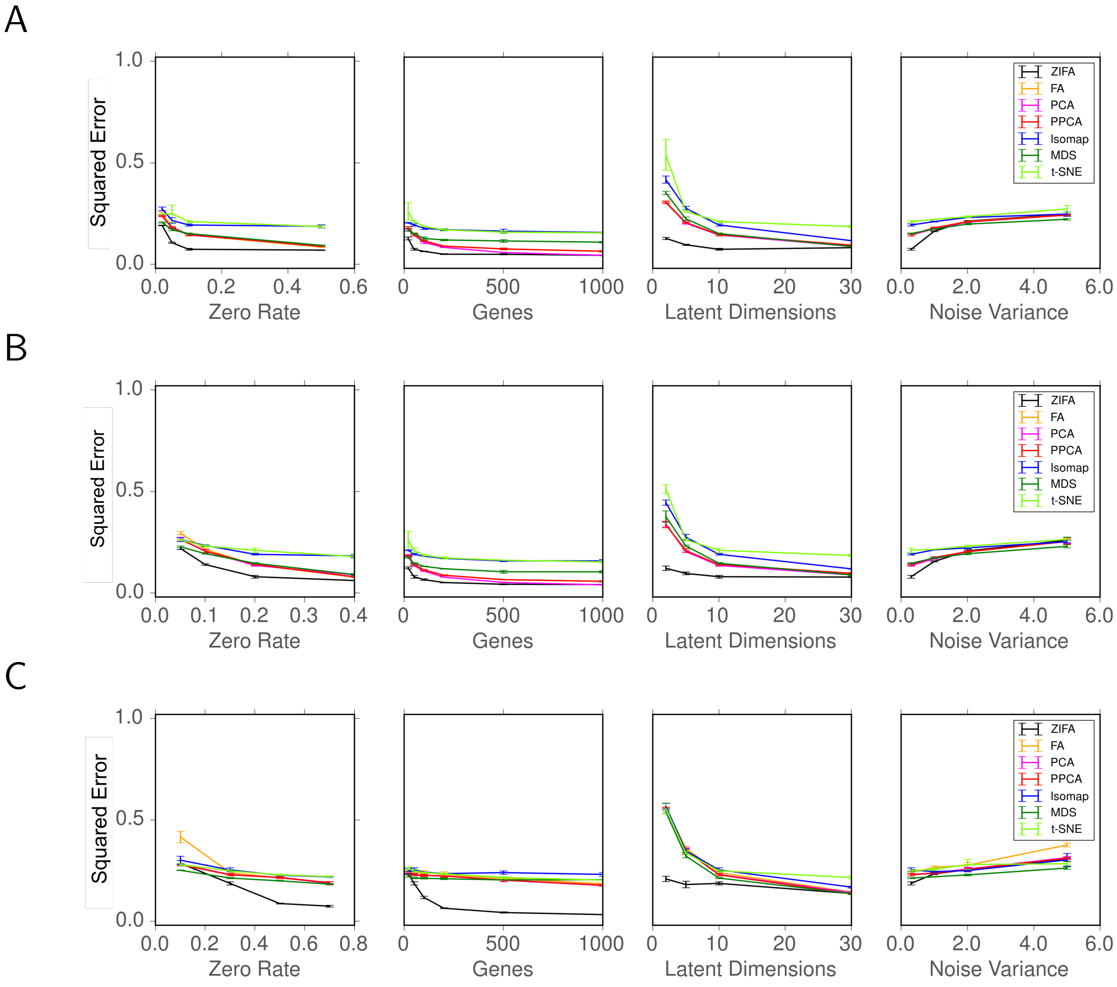

Figure 4: Performance of dimensionality reduction methods on simulated data using a squared-error metric, with lower scores denoting superior performance: (A) double-exponential decay, (B) linear decay $1 - \lambda\mu_0$ and (C) missing at random $1 - \lambda$ zero-inflation models.

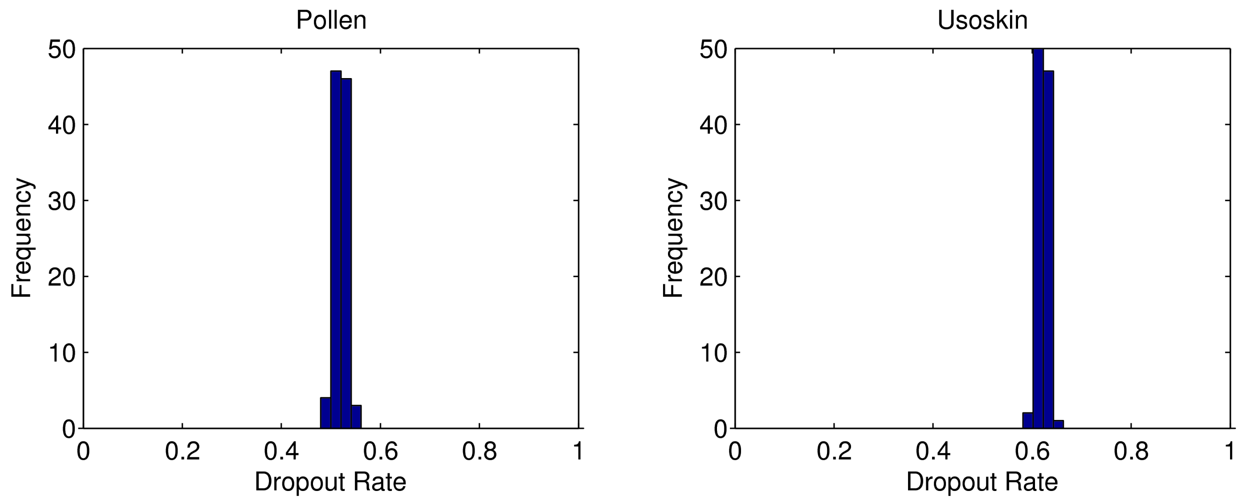

Figure 5: Distribution of average dropout rates over the random 500-gene subsets sampled from the Pollen [1] and Usoskin [2] data sets.
